# Oral Wild-Type *Salmonella* Typhi Challenge Induces Activation of Circulating Monocytes and Dendritic Cells in Individuals Who Develop Typhoid Disease

**DOI:** 10.1371/journal.pntd.0003837

**Published:** 2015-06-11

**Authors:** Franklin R. Toapanta, Paula J. Bernal, Stephanie Fresnay, Thomas C. Darton, Claire Jones, Claire S. Waddington, Christoph J. Blohmke, Gordon Dougan, Brian Angus, Myron M. Levine, Andrew J. Pollard, Marcelo B. Sztein

**Affiliations:** 1 Center for Vaccine Development, University of Maryland School of Medicine, Baltimore, Maryland, United States of America; 2 Department of Medicine, University of Maryland School of Medicine, Baltimore, Maryland, United States of America; 3 Department of Pediatrics, University of Maryland School of Medicine, Baltimore, Maryland, United States of America; 4 Oxford Vaccine Group, Department of Paediatrics, University of Oxford and the National Institute for Health Research (NIHR) Oxford Biomedical Research Centre, Oxford, United Kingdom; 5 Microbial Pathogenesis Group, Wellcome Trust Sanger Institute, Hinxton, United Kingdom; 6 Nuffield Department of Medicine, University of Oxford, Oxford, United Kingdom; Oxford University Clinical Research Unit, VIETNAM

## Abstract

A new human oral challenge model with wild-type *Salmonella* Typhi (*S*. Typhi) was recently developed. In this model, ingestion of 104 CFU of *Salmonella* resulted in 65% of subjects developing typhoid fever (referred here as typhoid diagnosis -TD-) 5–10 days post-challenge. TD criteria included meeting clinical (oral temperature ≥38°C for ≥12h) and/or microbiological (*S*. Typhi bacteremia) endpoints. One of the first lines of defense against pathogens are the cells of the innate immune system (e.g., monocytes, dendritic cells -DCs-). Various changes in circulating monocytes and DCs have been described in the murine *S*. Typhimurium model; however, whether similar changes are present in humans remains to be explored. To address these questions, a subset of volunteers (5 TD and 3 who did not develop typhoid despite oral challenge -NoTD-) were evaluated for changes in circulating monocytes and DCs. Expression of CD38 and CD40 were upregulated in monocytes and DCs in TD volunteers during the disease days (TD-0h to TD-96h). Moreover, integrin α4β7, a gut homing molecule, was upregulated on monocytes but not DCs. CD21 upregulation was only identified in DCs. These changes were not observed among NoTD volunteers despite the same oral challenge. Moreover, monocytes and DCs from NoTD volunteers showed increased binding to *S*. Typhi one day after challenge. These monocytes showed phosphorylation of p38MAPK, NFkB and Erk1/2 upon stimulation with *S*. Typhi-LPS-QDot micelles. In contrast, monocytes from TD volunteers showed only a moderate increase in *S*. Typhi binding 48h and 96h post-TD, and only Erk1/2 phosphorylation. This is the first study to describe different activation and migration profiles, as well as differential signaling patterns, in monocytes and DCs which relate directly to the clinical outcome following oral challenge with wild type *S*. Typhi.

## Author Summary

Typhoid fever continues to be a public health problem and novel more effective vaccines are needed. One of the limitations in the development of new vaccines is an incomplete understanding of the host-pathogen interactions. To gain new insights into these interactions a new human oral challenge model with wild-type *Salmonella* Typhi (*S*. Typhi) was recently developed. In this model, 65% of the challenged subjects developed typhoid fever (referred here as typhoid diagnosis-TD-). Monocytes and dendritic cells (DCs) are part of the innate immune system and one of the first lines of defense against pathogens. The changes induced in these cells by *S*. Typhi infection were studied in a subset of volunteers (5 TD and 3 who did not develop TD despite the same oral challenge-NoTD-). Monocytes and DCs showed upregulation of different activation molecules between TD and NoTD volunteers. Furthermore, monocytes from NoTD volunteers showed enhanced *S*. Typhi binding and activation of signaling pathways associated with the pattern recognition receptor (PRR) TLR4, one day after challenge. In contrast, monocytes from TD volunteers had a moderate increase in *S*. Typhi binding and different signaling profiles. Therefore, multiple differences in monocytes and DCs from TD and NoTD volunteers following wild type *S*. Typhi challenge were identified.

## Introduction

Typhoid fever caused by *Salmonella enterica* serovar Typhi (*S*. Typhi), a human-restricted pathogen, continues to be a major global public health problem [1, 2]. Due in part to the absence of a suitable animal model, several aspects of the human response to infection remain to be explored [3, 4]. Over forty years ago, a successful human challenge model, which helped to address some aspects of the host-pathogen interaction, was developed at the University of Maryland [3, 5]. In that model volunteers ingested *S*. Typhi (10^7^ colony-forming units (CFU) of wild-type *S*. Typhi) suspended in 45 mL of milk (without sodium bicarbonate) and 50% of participants developed clinical infection [3]. Data obtained from this model were critical for the development of vaccines (Ty21a), diagnostics and treatment [6–10]. A new controlled human infection model of *S*. Typhi was recently developed at the Centre for Clinical Vaccinology and Tropical Medicine, University of Oxford. In this novel outpatient model, participants challenged with 10^4^ CFU of *S*. Typhi (Quailes strain) in a sodium bicarbonate buffered solution resulted in 65% of participants being diagnosed with typhoid fever (referred here as typhoid diagnosis-TD-) [11].

One of the first lines of defense against pathogens are the cells of the innate immune system, including macrophages and dendritic cells (DCs). Circulating monocytes, derived from bone marrow precursors, show great plasticity and are able to differentiate into various types of macrophages and DCs as they migrate to tissues or when exposed to the appropriate cytokine environment [12–15]. *S*. Typhi in an intracellular pathogen that initially gains access to the host by the oral route. Several aspects of the pathogenesis of *Salmonella* have been elucidated in the mouse model using *S*. Typhimurium, which replicates some aspects of human typhoid disease (typhoid fever-like disease in mice) [16, 17]. For example, it has been demonstrated that *S*. Typhimurium invades M and epithelial cells of the gut. Subsequently, the bacteria pass through Peyer’s patches (PP), mesenteric lymph nodes (MLN), lymphatic vessels and the blood stream [18, 19]. Monocytes, most likely migrating from peripheral blood, have been shown to accumulate in PP and MLN following infection with *S*. Typhimurium [20]. DCs have been proposed to play an important role in the pathogenesis of *S*. Typhimurium by transporting the bacteria from the intestine to MLN and other lymphoid tissues [21]. The complement system is likely to enhance bacterial phagocytosis through opsonization mediated by C3b and C3d, which has been proposed as one of the mechanisms that allows *Salmonella* to reach an intracellular niche within macrophages and polymorphonuclear cells [22]. In rodents it has been demonstrated that the control of *S*. Typhimurium growth in the early phases of a primary infection requires reactive oxygen intermediates generated via the phagocyte NADPH oxidase [23], which is present in macrophages. Despite these advances in the role that monocytes, macrophages and DCs play in the murine typhoid model, whether similar mechanism(s) are operational in human typhoid disease remain to be elucidated. Evaluation of these phenomena in humans has been impaired since specimens from humans infected with wild-type (wt) *S*. Typhi immediately upon infection are exceedingly difficult to obtain and 40 years ago, when the last human challenges with wt *S*. Typhi were performed, the technology was not in place to evaluate these cells in detail. The development of a new human infection model of typhoid fever has provided a unique opportunity to begin exploring important questions about the role of circulating monocytes and DCs in this disease. In the current study we report changes in activation, migration and affinity for the pathogen in circulating monocytes of participants with typhoid diagnosis (TD) and those who did not developed disease (NoTD) following wild-type challenge with *S*. Typhi.

## Methods

### Human volunteers and clinical trial description

Samples used in this study were obtained as part of a clinical trial aimed at establishing a human model of *S*. Typhi infection. The study was performed at the Centre for Clinical Vaccinology and Tropical Medicine, University of Oxford, United Kingdom. Details of the clinical outcomes of this challenge model have already been published [11]. In short, healthy adults 18–60 years-old with no previous history of typhoid vaccination or residence (>6 months) in endemic areas were included. *S*. Typhi (Quailes strain) inocula were freshly prepared prior to each challenge by defrosting and suspending the required number of bacteria in 30 mL/0.53 g NaHCO_**3**_(aq). Participants fasted for 90 minutes before ingesting 120 mL/2.1 g NaHCO_**3**_(aq). Two minutes later, participants ingested the prepared challenge suspension. Volunteers were challenged either with 10^**3**^ or 10^**4**^ CFU of *S*. Typhi. Typhoid diagnosis included meeting clinical (temperature ≥ 38°C sustained for ≥ 12 hours) and/or microbiological (blood culture confirmed *S*. Typhi bacteremia) endpoints. Participants were reviewed daily for at least 14 days, recording the duration and severity of all solicited and unsolicited symptoms experienced and daily oral temperature readings (2 times per day). Indications for antibiotic treatment (ciprofloxacin, 500 mg twice daily, 14 days) included reaching typhoid diagnosis, unmanageable symptoms, or clinical necessity. All participants who did not develop typhoid received antibiotic treatment at day 14. Subsequent visits were performed at 21 and 28 days after challenge. In the current report we describe the results from a subset of volunteers challenged with 10^**4**^ CFU of *S*. Typhi. In this subset (n = 8), 5 volunteers were diagnosed with typhoid disease (TD) and 3 were not (despite a similar inoculum-NoTD-). In this exploratory study, specimens from a random subset of *S*. Typhi challenged participants were selected based on the number of existing peripheral blood mononuclear cells (PBMC) and the availability of as many of the critical time points as possible to enable a comprehensive evaluation of innate immune responses.

### Ethics statement

The clinical portion of the study was performed at the Centre for Clinical Vaccinology and Tropical Medicine, University of Oxford, United Kingdom [11]. Written informed consent was obtained from all volunteers and the procedures approved by the Oxfordshire Research Ethics Committee A (10/H0604/53) and the trial registered on the UK Clinical Research Network (identifier UKCRN ID 9297). Additionally, PBMC collected from healthy adult volunteers, recruited from the Baltimore-Washington area and University of Maryland, Baltimore campus, were also used in this study to characterize monocytes and DCs. Written informed consent was obtained from all volunteers and the procedures approved by the University of Maryland, Baltimore IRB (HCR-HP-00040025-6).

### Isolation of peripheral blood mononuclear cells (PBMC)

PBMC were isolated from blood samples collected from volunteers challenged with *S*. Typhi (Quailes strain). Sample collection was performed before challenge and at various time points thereafter. The evaluated time points differed slightly between TD and NoTD volunteers. These included day 0 (pre-challenge) and days 1, 2, 4, 7, 9, 14, 21 and 28 after challenge in all subjects. However, in TD volunteers extra samples were collected at the time of typhoid diagnosis (6–9 days after challenge with 10^**4**^ CFU of wt *S*. Typhi, [11]) as well as 48 and 96 hours later. PBMC were isolated immediately after blood draws by density gradient centrifugation and cryopreserved in liquid nitrogen following standard techniques [24].

### Cell surface staining for flow-cytometry

Cryopreserved PBMC were thawed and allowed to rest overnight at 37°C, 5% CO_2_ in complete media [RMPI (Gibco, NY, USA) supplemented with 10% fetal bovine serum (FBS) (Gemini Bioproducts, West Sacramento, CA), 2 mM L-glutamine (Gibco, Grand Island, NY, USA), 1x non-essential amino acids (Gibco, Grand Island, NY, USA), 10 mM HEPES (Gibco, Grand Island, NY, USA), 2.5 mM Sodium pyruvate, (Lonza, Walkersville, MD, USA), 100 U/ml Penicillin, 100 ug/ml streptomycin (Sigma-Aldrich, St. Louis, MO, USA), 50 μg/ml Gentamicin (Gibco, Grand Island, NY, USA)]. Cells were then harvested and stained for flow-cytometry in V-shaped 96-well plates using methods previously described [24, 25]. Briefly, 1x10^6^ cells were plated and stained for viability (20 min on ice) using 100 μl of Alexa Fluor 700-succinimidyl ester dye (0.4 μg/ml) (Invitrogen, USA). After 2 washes with flow cytometry staining (FC) buffer (4% FCS, 1X phosphate buffered saline (PBS) and 0.02 Sodium Azide), *S*. Typhi (strain Ty2) labeled with Pacific Blue dye (*S*. Typhi-PB) were added. Bacteria were labeled as previously described [26] and used at a 50:1 (bacteria:cells) ratio (20 min on ice). Cells were washed twice with FC buffer and then blocked with a mixture of mouse IgG (25 μl of a 200 μg/ml solution in FC buffer) (Meridian Life Sciences, Memphis, TN, USA) and human IgG (25 μl of a 1 mg/ml solution in FC buffer) (Sigma, St Louis, MO) for 10 minutes on ice. Cells were then stained with various antibody cocktails prepared in FC buffer. Monoclonal antibodies (mAbs) against the following molecules were used: CD19-ECD (clone J3-119; Beckman Coulter-BC-), CD38-PE-Cy5 (clone LS1298-4-3; BC), CD14-QDot 655 (clone TuK; Invitrogen), CD21-BV711 (clone B-ly4; BD), integrin α4β7-Alexa647 (clone ACT-1; Millennium, The Takeda Oncology Co), CD3-Alexa Fluor 700 (clone UCHT1; BD), CD66b-FITC (clone G10F5; BD), CD16-PE (clone 3G8; BD), HLA-DR (clone G46-6; BD), CD56 (clone B159; BD), CD11c-FITC (clone B-ly6; BD), CD123-PE (clone 9F5; BD), CD1c-APC (BDCA1; clone AD5-8E7; Miltenyi Biotec-MB-), CD303-Biot (BDCA2; clone AC144; MB) and CD141-VioBlue (BDCA3; clone AD5-14H12; MB). After washing cells twice with FC buffer, Pacific Orange-Streptavidin was added to each well (Invitrogen, USA). Stained cells were fixed with 1% PFA in PBS.

### Phosphoflow assay

#### Stimulants used

PBMC were stimulated with *S*. Typhi-LPS-QDot655 micelles of nanoparticle size (approx. 30–60 nm) [27]. To generate LPS-micelles, a previously published methodology was used [27–29]. In short, decane was evaporated from 100 μl of organic Quantum dots (QDots) 655 Innovator’s Tool Kit (ITK) (Invitrogen, Eugene, Oregon) using a speed vacuum concentrator. QDots were then re-suspended in 500 μl of chloroform. LPS from *S*. Typhi (1 mg re-suspended in 100 μl of endotoxin free water) (Sigma-Aldrich, St. Louis, MO) was then added, followed by methanol (dropwise) until a homogenous mix was achieved. Methanol was then allowed to evaporate from the LPS-QDot mix and re-suspended in 100 μl of endotoxin free water. The pH of the solution was basificated (pH 11) by adding a Tetramethylammonium hydroxide pentahydrate solution (100 mM) dropwise. The solution was then sonicated for 30 minutes and passed through two consecutive Zeba desalting columns (Pierce, Rockford, IL) to remove salts and excess of free LPS. LPS-micelles were collected in a total volume of 200 μl. Formation of LPS-micelles was determined by flow cytometry and the LPS concentration by LAL Chromogenic Endotoxin Kit (Pierce, Rockford, IL). PBMC were stimulated with approximately 5 μg of LPS. As a positive stimulation control 6 mM H_2_O_2_, a general phosphatase inhibitor, was used. 1% BSA in PBS was used as a negative stimulation control.

#### Cell stimulation and staining

Thawed PBMC were allowed to rest overnight in complete media at 37°C, 5% CO_2_. Cells were then plated in 12x17 mm tubes (1x10^6^ per reaction) and stained for viability using a yellow-fixable dye (Invitrogen, USA) (20 min on ice). After 2 washes with 1% BSA in PBS cells were re-suspended in 50 μl of ice cold 1% BSA and allowed to rest for at least 15 minutes. During this period stimulants were prepared freshly in 1% BSA in PBS. Stimulant aliquots (50 μl) were maintained at room temperature and added to the chilled PBMC. Cultures were then immediately transferred to a water bath (37°C) for 10 minutes. After stimulation, cells were fixed with 1 ml of 1.6% PFA at 37°C (5–10 min) and washed twice with FC buffer. Fixed cells were permeabilized with 80% ice cold methanol (1 ml) for 20 minutes at -20°C. To rehydrate PBMC, samples were washed twice with FC buffer. Samples were then blocked with a mixture of human and mouse IgG as described above and subsequently stained with mAb cocktails in FC buffer for 1 hour on ice in the dark. mAbs in the cocktails included: CD27-PE (clone L128; BD), pAKT-S473-Ax647 (clone D9E; Cell Signaling Technologies), CD20-PerCP-Cy5.5 (clone H1; BD), p38MAPK-T180/Y182-Pacific Blue (clone 36/p38 (pT180/pY182); BD), Erk1/2-T202/Y204-PE-CF594 (clone 20A; BD), p38MAPK-T180/Y182- PE-CF594 (clone 36/p38 (pT180/pY182), Btk-Y551-Alexa647 (clone BtkY551 & ItkY511; BD) and/or NFκB p65-pS529-PE-Cy7 (clone K10-895.12.50 (pS529; BD). After incubation cells were washed twice with FC buffer, fixed with 1% PFA in 1X PBS/EDTA and samples collected in a custom LSRII flow cytometer analyzer (Beckton-Dickson, USA). Samples were analyzed using FlowJo (Tree Star, San Francisco, CA) and Cytobank (Palo Alto, CA) software packages.

### Statistical methods

The percentages of monocytes and DCs in TD and NoTD volunteers before challenge were compared using the Mann-Whitney test. In challenged volunteers who developed typhoid, the data was plotted relative to the time of diagnosis (TD). All volunteers were diagnosed with typhoid 5–10 days post-challenge (10^4^ CFU); however, since not all volunteers developed typhoid at the same time, the data was grouped in narrow time frames (e.g., -8 to -6) to facilitate analysis and interpretation of the data. In NoTD volunteers the data is presented as a time following challenge. Changes in the expression of markers pre-challenge and post-challenge (at different time frames) were evaluated by one way ANOVA followed by a Dunnett’s post-test using pre-challenge as the comparator. For comparison of the groups of cells expressing multiple markers a one way ANOVA followed by a Bonferroni’s multiple comparison test was done. Statistical analysis was performed in GraphPad Prism software (GraphPad Software Inc., USA). All hypotheses were evaluated using two-sided tests. Two-sided p values <0.05, without adjustment for multiple comparisons, were considered statistically significant.

## Results

### Changes in activation and gut homing markers induced by *S*. Typhi in circulating monocytes

**Fig 1:**
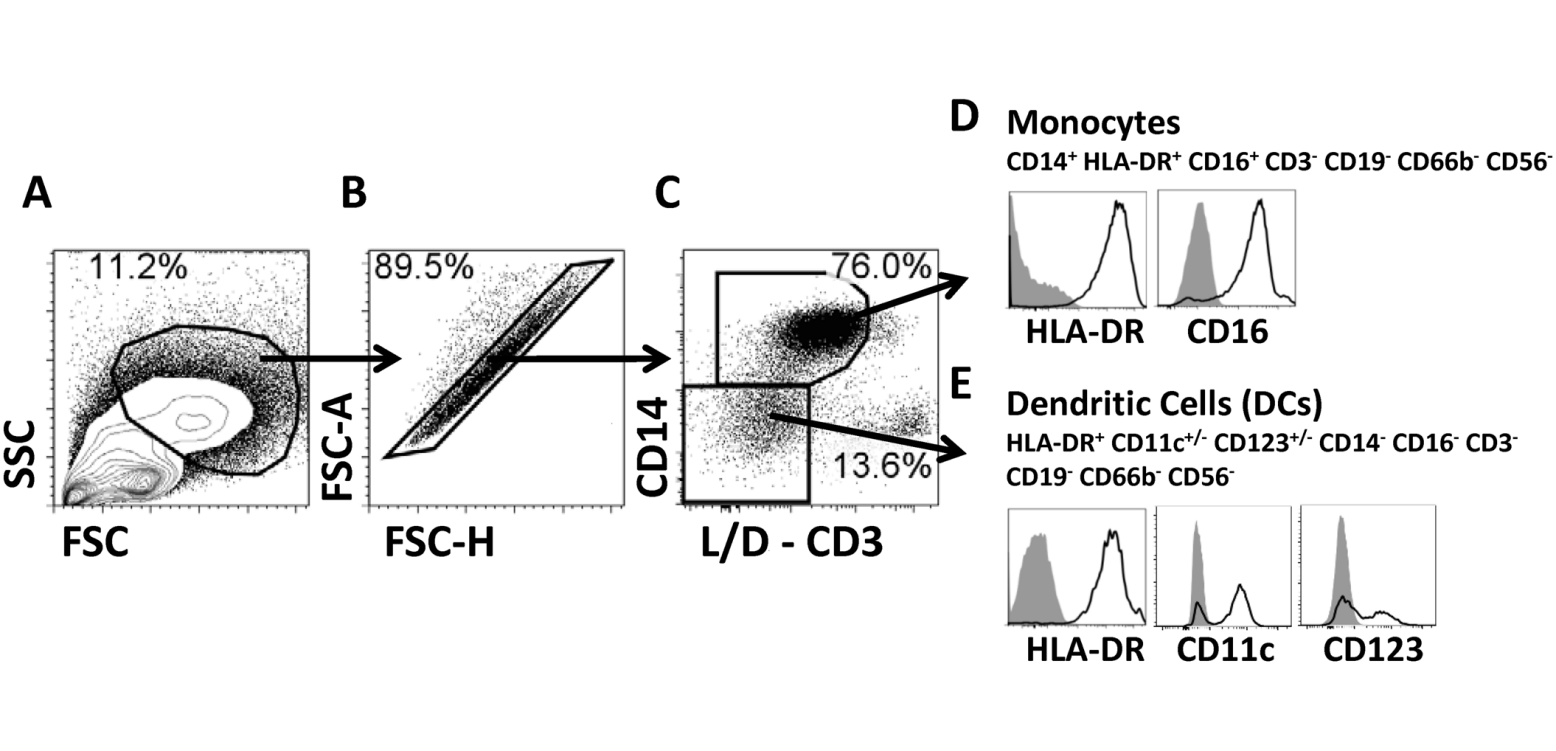
Gating strategy. Stained PBMC were gated on cells with high FSC and SSC characteristics avoiding the lymphocyte region (A), doublets/multiplets were eliminated (B) and subsequently live cells were gated based on expression of CD14 (C). The viability channel (live/dead-L/D-) also contained CD3; therefore cells selected were also CD3^-^ (C). CD14+ cells also expressed CD16 and HLA-DR. Therefore, these cells were classified as monocytes (D). All CD3^-^CD14^-^ expressed HLA-DR and a percentage of these cells expressed either CD11c or CD123 but lacked CD19, CD66b, CD16 and CD56; therefore, these cells were classified as dendritic cells (DC) (E) (see also S4 Fig). The identity of these cells as DCs was confirmed using PBMC from healthy volunteers stained with monoclonal antibodies to BDCA-1, BDCA-2 and BDCA-3 (S4 Fig). Plots shown are from a representative volunteer. Gray histograms represent fluorescent minus one (FMO) samples.

**Fig 2:**
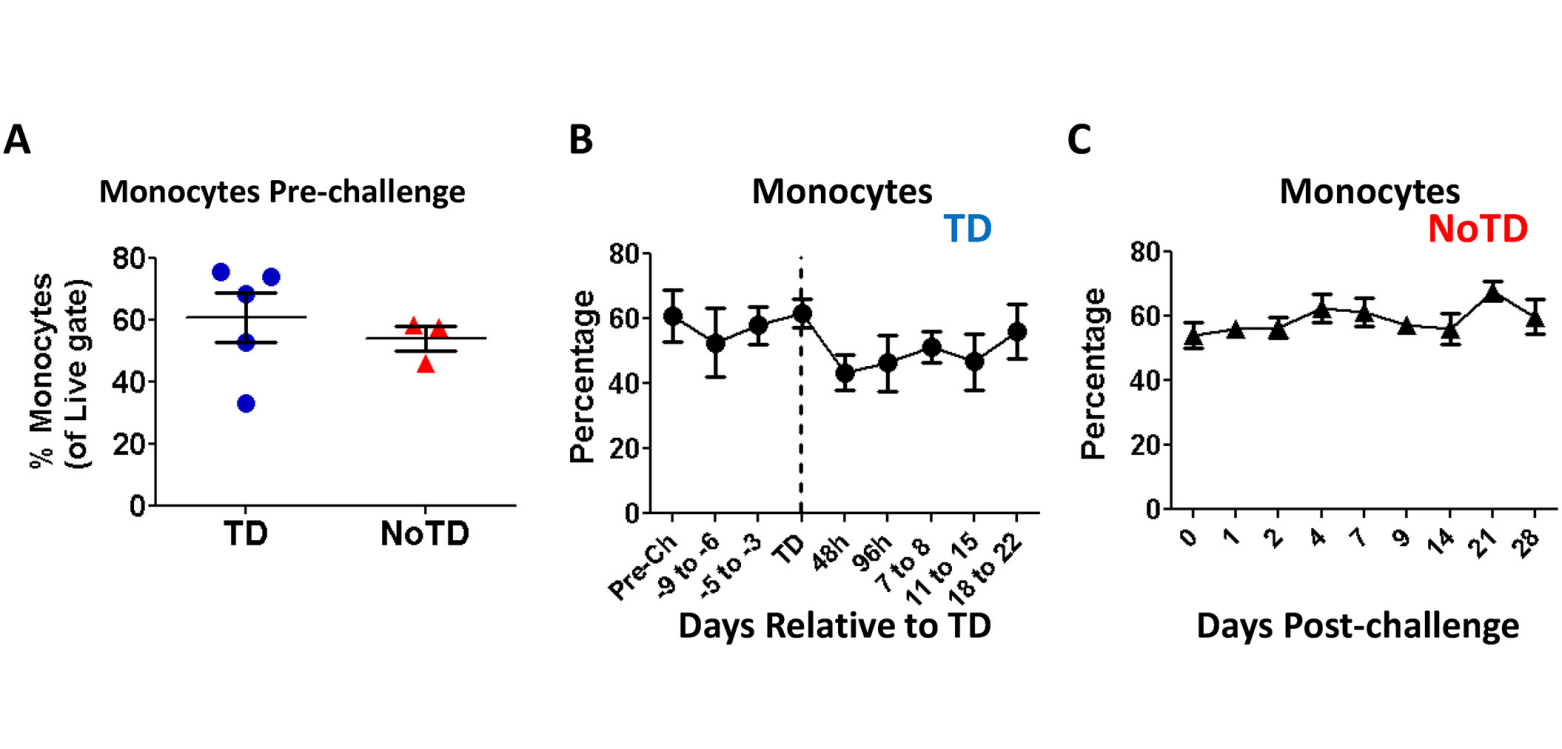
Monocyte levels in peripheral blood following infection with wt *S*. Typhi. The percentages of circulating monocytes in TD (circles; n = 5) and NoTD (triangles; n = 3) participants were compared to the levels before wt *S*. Typhi challenge (**A**), as well as in subsequent days (**B** and **C**). In TD volunteers (**B**), the data is depicted relative to the time of typhoid diagnosis (TD) (dotted vertical line). Time points before (except for pre-challenge) and after (>+7 days) TD were depicted in narrow time frames whilst TD, TD-48h and TD-96h are shown as individual time points. Mean ± SE are presented in all graphs.

Monocytes (CD14^**+**^ CD16^**+**^ HLA-DR^**+**^ CD56^-^ CD66b^-^ CD3^-^ CD19^-^) were identified in the non-lymphocytic region of the FSC/SSC bi-exponential plots (Fig 1). No significant differences were observed in the percentages of monocytes recorded before *S*. Typhi challenge between participants who developed TD and those who did not (NoTD) upon challenge with wt *S*. Typhi (Fig 2A). We next assessed changes in the percentage of monocytes after challenge. No changes were observed in TD volunteers until 2 days after typhoid diagnosis (TD-48h), when a small reduction in the percentage of these cells was noted (Fig 2B). The reductions in the percentages of monocytes appeared to persist for several days; however, the differences compared to pre-challenge were not statistically significant. The frequency of these cells began to return to baseline levels 18 days after diagnosis. In contrast, no changes in the frequency of monocytes following challenge were recorded in NoTD volunteers (Fig 2C). Interestingly, the number of circulating monocytes measured in whole blood (whole blood cell counts-WCC-) [11] did not show significant changes compared to pre-challenge levels in either TD or NoTD participants, although a small decrease was observed in TD participants starting 8 days after diagnosis (S1A and S1B Fig).

**Fig 3:**
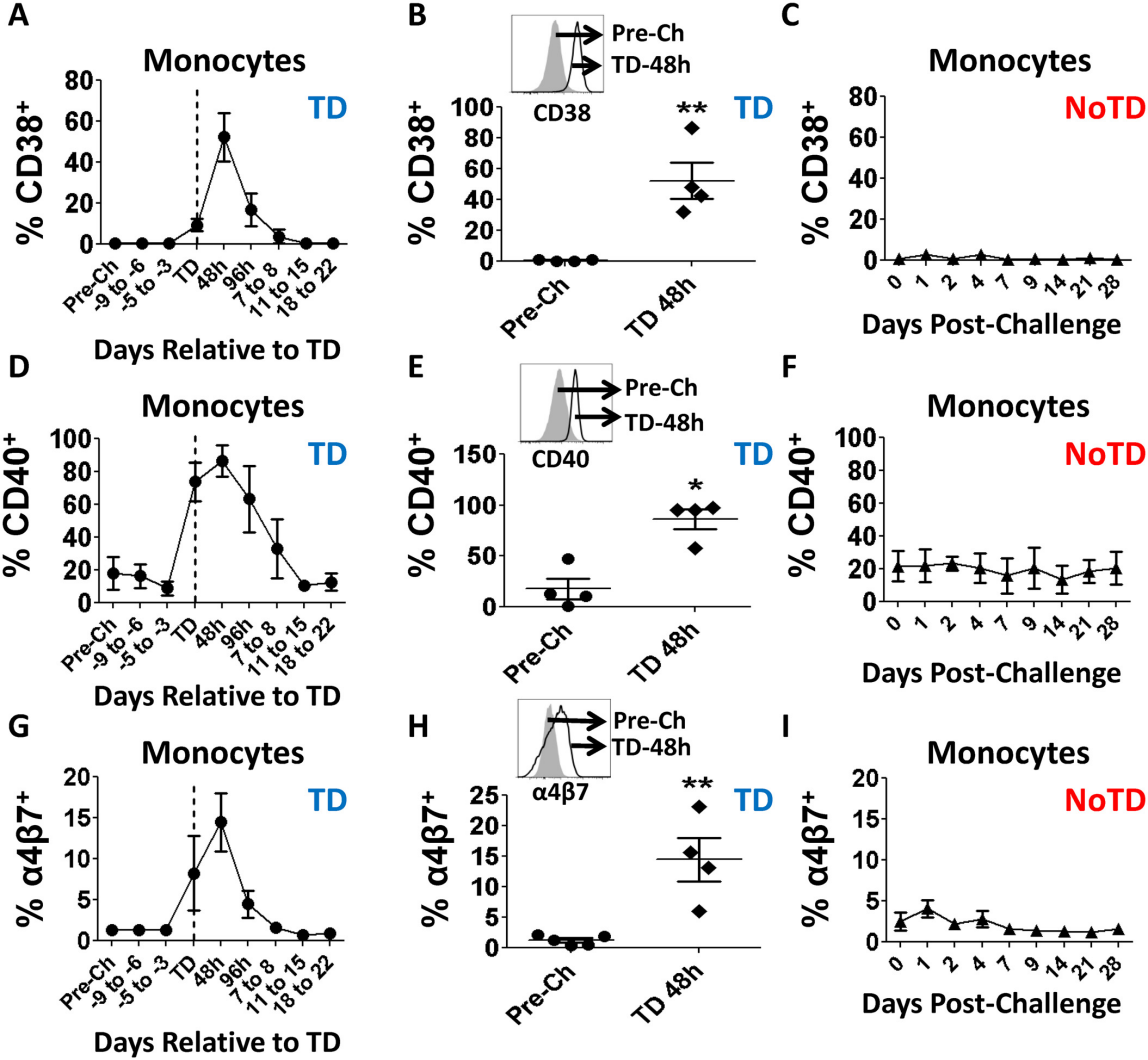
Changes in CD38, CD40 and integrin α4β7 expression in monocytes following wt *S*. Typhi challenge. Shown in panel **A**, **D** and **G** are time courses of the changes in CD38, CD40 and integrin α4β7 expression, respectively, in TD volunteers. Shown in panels **B**, **E** and **H** are the data in individual volunteers at pre-challenge (circles) and peak up-regulation (TD-48h) (black diamonds) for these markers in TD volunteers. The histogram insert contains an example of the up-regulation of each marker in a representative volunteer. Shown in panels **C**, **F** and **I** are the time courses of the expression of CD38, CD40 and integrin α4β7 in NoTD volunteers. Statistical significance compared to pre-challenge is indicated by: * p<0.05; **p<0.005 (Dunnett’s multiple comparison test).

To determine levels of activation of monocytes, changes in the expression of CD38 and CD40 were evaluated (Fig 3). TD volunteers showed an increase in the expression of CD38 and CD40 at the time of typhoid diagnosis or in subsequent days (Fig 3A and 3D). Both markers showed a peak up-regulation at TD-48h, which was statistically significant, compared to pre-challenge levels (Fig 3B and 3E). In subsequent days, CD38 and CD40 expression returned to baseline levels (Fig 3A and 3D). In contrast, no changes in these markers were observed in NoTD volunteers (Fig 3C and 3F). Furthermore, changes in the expression of integrin α4β7 following challenge were evaluated to determine whether these activated monocytes have the potential to migrate to the gut. Similar to observations with CD38 and CD40, TD volunteers up-regulated this marker during disease reaching peak levels TD-48h, at which time point the differences were statistically significant compared with pre-challenge (Fig 3G and 3H). No changes in the expression of this marker were identified in NoTD volunteers (Fig 3I).

### Ability of monocytes to bind *S*. Typhi and activation of signaling pathways

**Fig 4:**
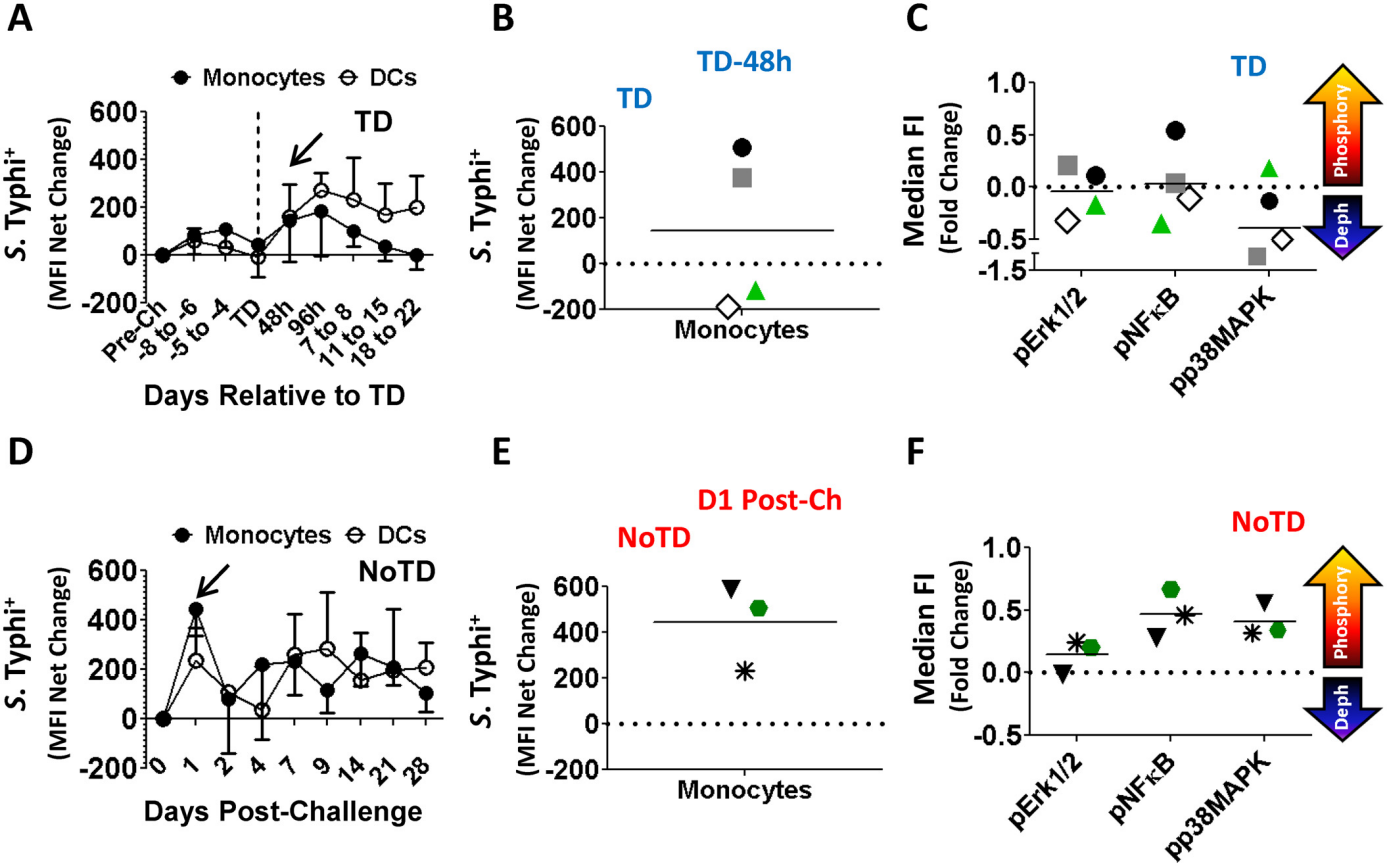
Ability of monocytes to bind *S*. Typhi and activation of signaling pathways following wild-type challenge. Time courses of *S*. Typhi (killed-fluorescently labeled) binding to circulating monocytes from TD (**A**) and NoTD (**D**) volunteers. Shown in panels **B** and **E** are the binding data of individual volunteers at the peak time for TD (TD-48h) and NoTD (D1 Post-challenge) participants, respectively. Each volunteer is indicated by a defined symbol and color, which is also used to represent the data in panels **C** and **F**. The horizontal solid lines indicate the mean. Shown in panels **C** and **F** are the phosphorylation profiles of Erk1/2, pNFκB and p38MAPK following stimulation with *S*. Typhi-LPS QD-655micelles in TD and NoTD volunteers, respectively, at peak avidity. The data is presented as fold changes at peak *S*. Typhi binding compared to pre-challenge. The dotted line indicates no changes in phosphorylation and the horizontal lines represent the mean.

We next evaluated the ability of circulating monocytes to bind *S*. Typhi. These key innate immune cells are likely to interact with *S*. Typhi LPS (the most abundant antigen on the surface of the bacteria) through TLR4, which is expressed by monocytes [30, 31]. Interaction of circulating monocytes with *S*. Typhi was evaluated by measuring changes in the relative abundance of the bacteria on the surface of the cells as determined by changes in mean fluorescence intensity (MFI) (Fig 4). In TD volunteers an increase in the interaction of monocytes with *S*. Typhi was apparent 48–96 hours after TD (TD-48h and TD-96h) (Fig 4A). It is of importance to notice that these are the same time points in which the other parameters evaluated also showed the most significant changes. Detailed analysis at TD-48h post-diagnosis in TD volunteers revealed that 2 of 4 volunteers showed increased ability to bind *S*. Typhi (Fig 4B). To evaluate if the increased binding of monocytes for *S*. Typhi resulted in increased activation of these cells, phosphorylation of signaling pathways associated with TLR4 [NFκB-p65 (pS529), Erk1/2 (T202/Y204) and p38MAPK (pT180/pY182)] were studied using *S*. Typhi LPS-micelles as the stimulant (10 min, 37°C). The results showed that in TD volunteers, the monocytes of the 2 volunteers with enhanced avidity for *S*. Typhi (peak at TD-48h) phosphorylated Erk1/2 (T202/Y204), only one of them NFκB-p65 (pS529), but none of them p38MAPK (pT180/pY182) (Fig 4C). In contrast, in NoTD volunteers a spike in the interaction with *S*. Typhi by monocytes was also noted; however, this was present immediately after challenge (D1) (arrow) (Fig 4D) and all the NoTD volunteers showed this increased interaction. Furthermore, monocytes of these volunteers phosphorylated all the signaling proteins evaluated (NFκB-p65, Erk1/2 and p38MAPK) at the peak interaction time with *S*. Typhi (D1 post-challenge) (Fig 4E).

### Changes in activation markers induced by *S*. Typhi in circulating DCs

**Fig 5:**
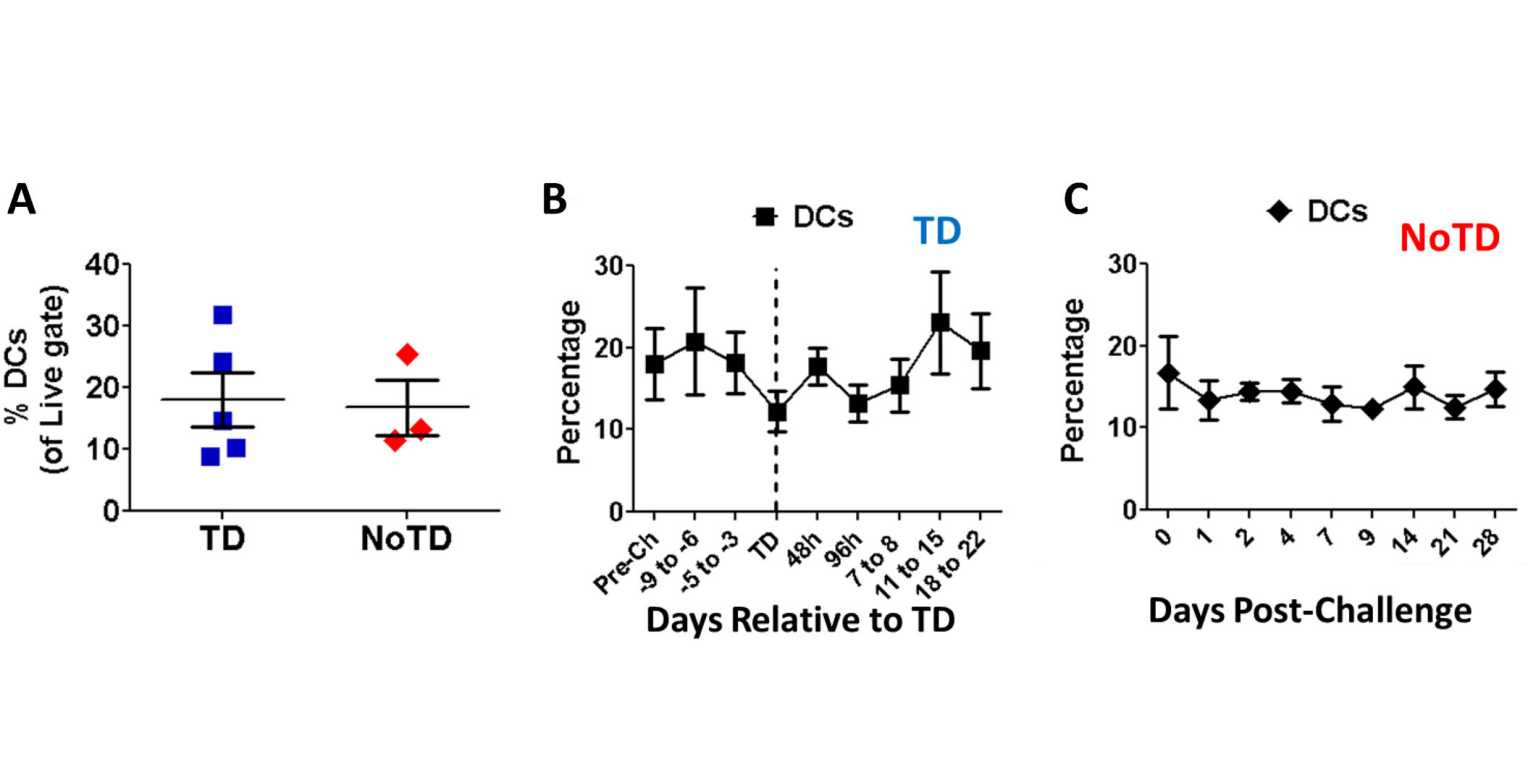
DCs levels in peripheral blood following infection with wt *S*. Typhi. The percentages of circulating DCs in TD (squares; n = 5) and NoTD (diamonds; n = 3) volunteers were compared to the levels before wt *S*. Typhi challenge (**A**) as well as in subsequent days (**B** and **C**). The graphs display mean ± SE.

**Fig 6:**
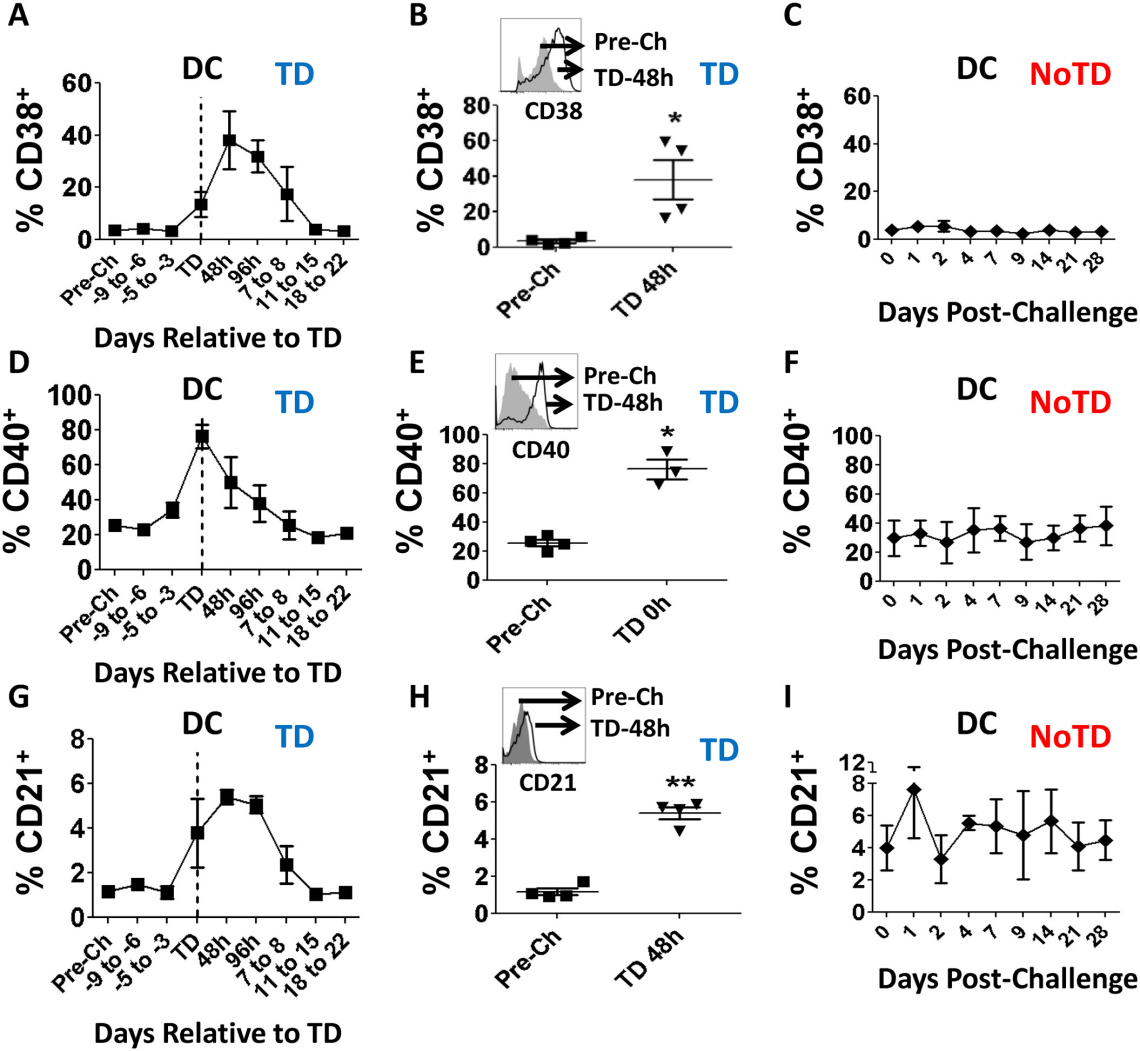
Changes in CD38, CD40 and CD21 expression in DCs following wt *S*. Typhi challenge. Shown in panels **A**, **D** and **G** are the time courses of the changes in CD38, CD40 and CD21 expression, respectively, in TD volunteers. Shown in panels **B**, **E** and **H** are the data in individual volunteers at pre-challenge (squares) and peak up-regulation times (TD-0h or TD-48h) (inverted triangles) for these markers in TD volunteers. The histogram insert contains an example of the up-regulation for each marker, in a representative volunteer. Shown in panels **C**, **F** and **I** are the time courses of expression of CD38, CD40 and CD21 in NoTD volunteers. Statistical significance compared to pre-challenge is indicated by: * p<0.05; **p<0.005 (Dunnett’s multiple comparison test).

Similar to monocytes, DCs (HLA-DR^+^ CD11c^+/-^ CD123^+/-^ CD14^-^ CD3^-^ CD19^-^ CD16^-^ CD56^-^ CD66b^-^) were identified in the non-lymphocytic region of the FSC/SSC bi-exponential plots (Fig 1). Furthermore, comparable to the observations with circulating monocytes, no differences in the percentage of DCs were observed in TD and NoTD participants before challenge with wt *S*. Typhi (Fig 5A). Additionally, neither TD nor NoTD volunteers showed changes in the percentage of DCs in the days after challenge (Figs 5B and 3C). In TD volunteers, the activation markers CD38 and CD40 were significantly up-regulated in DCs at the time of typhoid diagnosis or in the subsequent 96 hours (Fig 6A and 6D). The peak increase of these molecules was TD-48h and TD for CD38 and CD40, respectively, at which time point the differences were statistically significant compared to pre-challenge (Fig 6B and 6E). Up-regulation of these markers was not detected in DCs of NoTD volunteers (Fig 6C and 6F). We also evaluated changes in the expression of CD21 induced by *S*. Typhi challenge. Results showed that a relatively modest percentage of DCs in TD volunteers up-regulated this molecule after typhoid diagnosis. The peak up-regulation was observed at the TD-48h and TD-96h time points (Fig 6G and 6H). In contrast, CD21 expression, except for a modest increase 1 day after challenge, remained unchanged compared to pre-challenge in NoTD volunteers (Fig 6I). We also evaluated the gut migration potential of DCs by studying changes in the expression of integrin α4β7; however, no significant changes following challenge were detected in either TD or NoTD volunteers (S1C and S1D Fig).

### Ability of DC to bind *S*. Typhi

**Fig 7:**
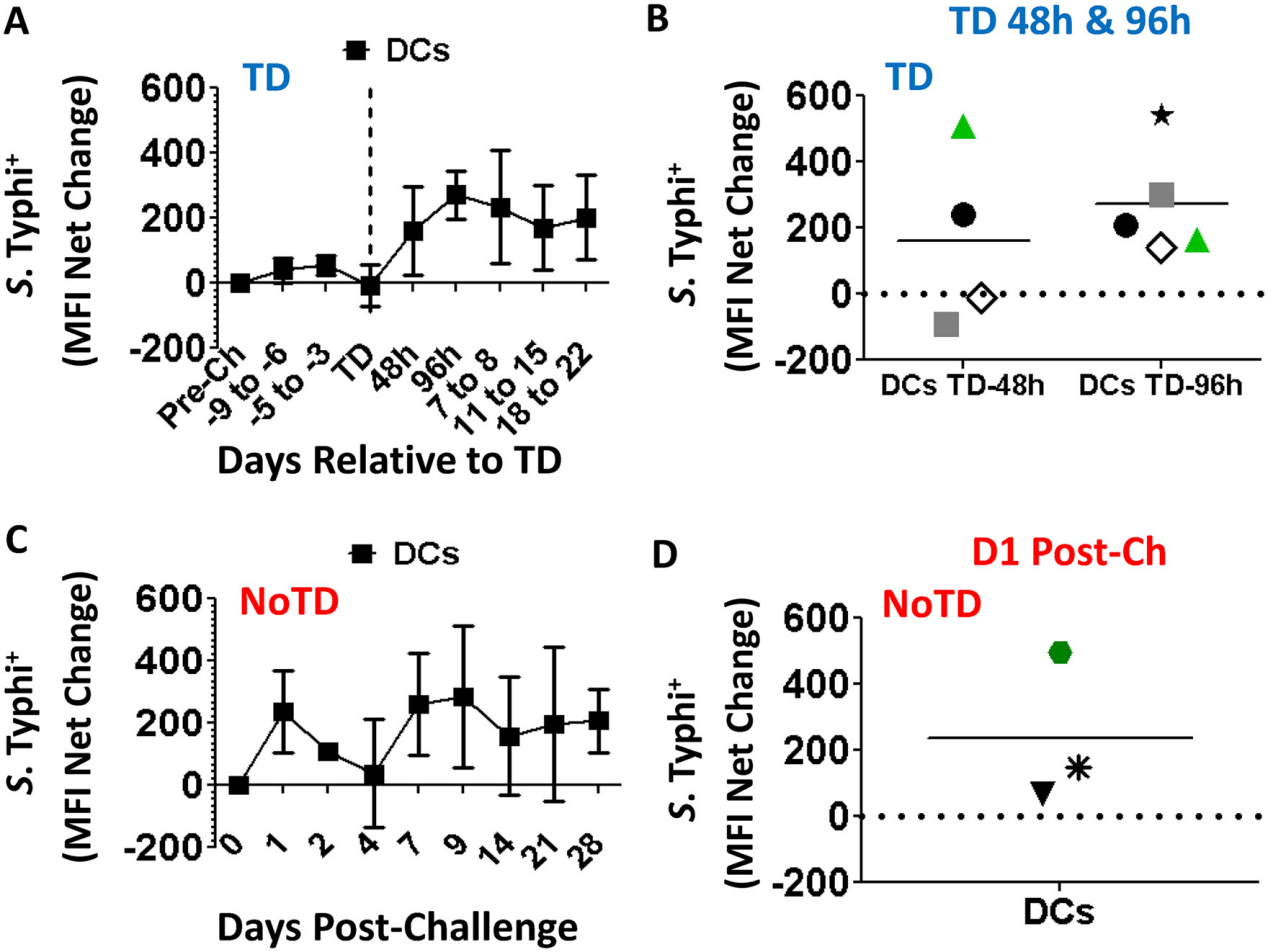
Ability of DCs to bind *S*. Typhi following wild-type challenge. Time courses of the binding ability of circulating DCs from TD (**A**) and NoTD (**C**) volunteers for *S*. Typhi (killed-fluorescently labeled). Shown in panels **B** and **D** are the data of individual volunteers at peak time of binding in TD (TD-48h and TD-96h) and NoTD (D1 Post-challenge) volunteers, respectively. Each volunteer is indicated by a defined symbol and color, as in Fig 4. The horizontal line indicates the means.

The ability of DCs to bind *S*. Typhi was evaluated as described above. In TD volunteers an increase in the interaction of *S*. Typhi with DCs was evident after typhoid diagnosis with a peak at TD-96h (Fig 7A). A more detailed analysis at this time point revealed that all 5 volunteers evaluated showed increased avidity for the bacteria (Fig 7B). Remarkably, in NoTD volunteers, as was observed in monocytes, a spike in the interaction with *S*. Typhi was present immediately after challenge (D1), with varying degrees depending on the individual participants (Fig 7D).

### Expression of gut homing activation markers is independent of *S*. Typhi binding

The dependence of the studied molecules, alone or in combination, on the binding to *S*. Typhi was explored in monocytes and DCs using boolean gates (S2 Fig). For this analysis, an arbitrary gate to define the percentage of cells binding to *S*. Typhi (>0.5%), was used. These gates were based on FMOs. These evaluations were performed only in samples from 48h post-TD, the time point in which most of the markers showed significant up-regulation. Only the 5 most prominent populations of 16 possible combinations, in monocytes and DCs are displayed (S2 Fig). These 5 populations account for >90% and >60% of the monocytes and DCs, respectively. In monocytes, the most abundant population was the one co-expressing CD38 and CD40, the frequency of these cells was significant higher (p<0.05) than the other ones, except for the one expressing CD40 alone. However, the predominance of CD40^+^CD38^+^ and CD40^+^ cells was independent of the binding to *S*. Typhi (S2A and S2B Fig), as indicated by the similar patterns identified between the cells that interacted with *S*. Typhi and the ones that did not. Interestingly, CD38^+^CD40^+^ and integrin α4β7^+^CD38^+^CD40^+^ populations were not statistically different in *S*. Typhi^-^ cells (S2B Fig). By contrast, in *S*. Typhi^+^ cells these populations showed statistical significance (S2A Fig). Finally, when the *S*. Typhi^+^ and *S*. Typhi^-^ cells were compared, integrin α4β7^+^CD38^+^CD40^+^, integrin α4β7^+^CD40^+^ and CD38^+^CD40^+^ populations were statistically higher in frequency in the *S*. Typhi^-^ group than in the *S*. Typhi^+^ one.

In DCs, in the *S*. Typhi^+^ cells (S2C Fig) the most abundant population was the one expressing CD40 alone followed by cells expressing CD38 alone. However, no statistical difference was found between the 5 populations evaluated. In *S*. Typhi^-^ DCs, the most abundant population was the one upregulating CD38 alone. There were no statistical differences between the populations evaluated in *S*. Typhi^-^ cells (S2D Fig); however, when compared to the *S*. Typhi^+^ cells, upregulation of CD38 alone was statistically significantly higher in *S*. Typhi^-^ cells (S2C and S2D Fig).

## Discussion

*S*. Typhi is a human restricted pathogen and no animal model is capable of reproducing all the clinical aspects of this disease. Therefore, studies in humans are necessary to understand pathogen-human host immune interactions which can ultimately aid in the design of novel vaccines. The specimens used in the current study were collected as part of a parent study aiming at developing a new human challenge model to study typhoid disease [11]. Macrophages are one of the first lines of defense against *Salmonella* and at the same time a primary target of infection by these microorganisms. However, several aspects of their *in-vivo* interaction remain unknown or poorly understood. In the current study we aimed at studying changes in frequency, activation, ability to bind *S*. Typhi and signaling induced by this microorganism in peripheral blood monocytes and DCs of volunteers challenged with 10^4^ CFU of wt *S*. Typhi (Quailes strain). Importantly, by comparing the results between TD and NoTD volunteers we aimed at furthering our understanding of the role of these important components of the human innate immune response when first encountering *S*. Typhi, and their potential to impact the development of disease.

No differences in the frequency of monocytes or DCs were identified before wild-type challenge, suggesting that it is not possible to predict which volunteers will develop disease by simply looking at the frequency of these cells in circulation (Figs 2A and 5A). Curiously, what appears to be a small reduction in the percentage of monocytes was observed immediately after typhoid diagnosis (TD), a finding consistent with the immunosuppression reported in animal models [32, 33] as well as with the decrease in white blood cell counts reported in the parent clinical trial [11]. However, the reductions in the percentages of monocytes as measured by flow cytometry or by WCC was not statistically different (S1A and S1B Fig). The most likely explanation for this phenomenon is that the “disappearance” of some of these cells from peripheral blood is due to their migration to the affected organs, such as the gut, as suggested by the up-regulation of integrin α4β7 (Fig 3G and 3H). The lack of changes in the percentage of DCs in TD volunteers, which correlates with the absence of changes in the expression of integrin α4β7 supports this hypothesis (Fig 5B and S1 Fig).

The minor fluctuations in the percentage of circulating DCs in TD volunteers (Fig 5B) initially suggested that these cells either played a discrete role in typhoid or responded in a different manner than monocytes. A more detailed analysis showed up-regulation of molecules associated with DC activation (CD38 and CD40) indicating that these cells are likely to play a role in typhoid (Fig 6A, 6B, 6D and 6E). Up-regulation of CD21 (Fig 6G and 6H) further confirms that DCs are activated in typhoid disease; however, the lack of changes in the expression of integrin α4β7 (S1C and S1D Fig) suggests that DCs perform their function(s) in a different organ than monocytes. Of note, CD21 is a molecule not usually associated with DCs, but with follicular dendritic cells, which are not present in circulation. However, *in-vitro* experiments have demonstrated that monocyte-derived DCs up-regulate this marker once they mature [34]. CD21 is the receptor for C3d, the final degradation product of the third component (C3) of complement; therefore, up-regulation of this marker suggests that a small group of DCs have an increased ability to bind opsonized bacteria, facilitating phagocytosis of the pathogen. This enhanced phagocytosis can have 2 consequences: (1) bacteria use this mechanism to disseminate into the blood stream [35] or (2) it is a mechanism of defense that ultimately leads to destruction of the bacteria in the phagosome by reactive oxygen species (ROS) and other reactive molecules. These consequences are not necessarily mutually exclusive, even though it is reasonable to speculate that in the former scenario the bacteria remain alive while in the latter, the bacteria are killed. However, even in the second scenario some bacteria may survive the effects of ROS, resulting in infective organisms which could spread to local lymph nodes and systemically. Taken together, up-regulation of CD38, CD40 and CD21 suggest that DCs play a role in typhoid disease. Nevertheless, the lack changes in expression of integrin α4β7 suggest that these cells perform their function in a different organ/lymphoid tissue than monocytes.

Up-regulation of CD38 in monocytes and DCs is dependent on the presence of IFN-γ [36]. Interestingly, increased levels of IFN-γ and IP-10 were observed in the serum from TD volunteers. The presence of these cytokines was first evidenced in some volunteers 96 h before typhoid diagnosis and elevated levels of IFN-γ were found in practically all the volunteers 48h post-diagnosis (Blohmke, C et al., in preparation). Importantly, recent reports suggest that CD38 synergizes with MHCII to enhance T cell proliferation [37], as well as CD83 expression and induction of IL-2 production [38]. Therefore, CD38 might play a role both in antigen presentation and modulation of T cell activation and expansion. Finally, CD38 is a molecule that has been associated with maturation of monocyte-derived DCs. Similar to CD38, CD40 was up-regulated in both monocytes and DCs; however, there were some notable differences in the observed kinetics, i.e., the peak expression was observed in DCs earlier (TD) than in monocytes (TD**-**48h). These differences, however, were not statistically significant (S3 Fig).

In monocytes, expression and/or co-expression of CD38, CD40 and integrin α4β7 appeared to be independent of bacteria binding since *S*. Typhi^+^ and *S*. Typhi^-^ monocytes showed a similar pattern of up-regulation of these markers (S2A and S2B Fig). The statistical differences identified in the populations between *S*. Typhi^+^ and *S*. Typhi^-^ monocytes are likely the result of the higher abundance of *S*. Typhi^-^ cells (6–8 fold higher). Therefore, these data suggest that in monocytes up-regulation of these molecules was more likely dependent on the cytokine milieu rather than on the direct interaction with the microorganism. In DCs, even though some differences in the patterns of the populations between *S*. Typhi^+^ and *S*. Typhi^-^ cells were identified (S2A and S2B Fig), no statistical differences were identified within *S*. Typhi^+^ (S2A Fig) or *S*. Typhi^-^ cells (S2B Fig). Therefore, it appears that, similar to monocytes, expression and/or co-expression of CD21, CD38 and CD40 is dependent of the cytokine environment and not on their interaction with *S*. Typhi.

In the case of B and T cells it has been well documented that the induction of integrin α4β7 is dependent on the interaction with intestinal DCs or stromal mesenteric lymph node cells capable of producing retinoic acid (RA). These cells reside in Peyer’s patches (PP) and/or mesenteric lymph nodes (MLN) [39–42]. Integrin α4β7-expressing B and T cells identified in peripheral blood are re-circulating cells that left PP or MLN upon being primed by intestinal DCs. However, little is known concerning the expression of integrin α4β7 in circulating monocytes, particularly in salmonellosis in humans. Circulating monocytes exhibit developmental plasticity and upon entering tissues they can differentiate into macrophages or monocyte-derived DCs [12, 14, 15, 43]. In our study, a major feature of circulating monocytes is their ability to up-regulate integrin α4β7 during the typhoid disease days (Fig 3G and 3H). Less clear, however, was the mechanism behind this up-regulation since monocytes reaching tissues will become terminally differentiated cells and are not expected to recirculate to peripheral blood. Therefore, our data suggest that another, not yet identified mechanism, is responsible for up-regulation of integrin α4β7 in monocytes. We can speculate that soluble factors (e.g., cytokine(s) or other small molecule(s)) are ultimately responsible for enhancing the expression of integrin α4β7 in monocytes during typhoid.

One of the most interesting findings is that monocytes and DCs in all NoTD volunteers showed a spike in their ability to bind *S*. Typhi almost immediately after challenge (D1) (Figs 4D and 7C). Remarkably, following *in-vitro* stimulation with *S*. Typhi-LPS, monocytes from all of these volunteers showed phosphorylation of NFκB and p38MAPK. Additionally, 2 of the volunteers phosphorylated Erk1/2 (Fig 4F). These three proteins are associated with the TLR4 signaling pathway. On the other hand, TD volunteers also showed a spike in their ability to bind *S*. Typhi; however, this occurred late in the infection and only in 2 of the 4 volunteers evaluated. Additionally, it appears that despite that these 2 volunteers showed increased binding of the bacteria, the signaling pathways induced were different from the NoTD volunteers since phosphorylation of p38MAPK was not detected and only one of the 2 phosphorylated NFκB. Unfortunately, this type of analysis was not possible in DCs, mainly due to technological limitations (lack of mAbs that allow proper resolution of the DC population). Taken together these findings suggest that the initial interaction of monocytes with *S*. Typhi and activation of the appropriate signaling pathways might limit the ability of this microorganism to cause disease. In the case of NoTD volunteers we can hypothesize that the bacteria were “neutralized” immediately after challenge, likely in the gut microenvironment, precluding progression to typhoid disease. Whether this is due to effective destruction of the microorganisms in the phagosome or other mechanism(s) remains to be explored. In the case of TD volunteers *S*. Typhi avoided neutralization following challenge and was able to establish an infection. It seems that monocytes from these volunteers have difficulty mounting an appropriate response as evidenced by their lack of an increased ability to bind *S*. Typhi early in infection and induction of different signaling pathways. In these volunteers, clearance of the disease most likely will rely more heavily on adaptive immune responses. It is important to notice that monocytes from TD volunteers with reduced binding of *S*. Typhi (48h) showed de-phosphorylation of Erk1/2, and NFκB (Fig 4C), which might indicate that phosphatases are particularly active in monocytes from these participants. Taken together, these results further support the idea that different signaling pathways are predominant in TD volunteers. We acknowledge, however, that since we evaluated phosphorylation at a single time point (10 minutes stimulation), we might be capturing a difference in the kinetics of phosphorylation and de-phosphorylation of the signaling proteins. Therefore, future studies will be directed to evaluate in more detail the kinetics of the signaling pathways associated with TLR4 activation in TD and NoTD volunteers.

In summary, in the current study we have demonstrated for the first time that circulating monocytes and DCs are activated following exposure to wt *S*. Typhi in humans. As expected, these cells showed different migration profiles and most likely functions during disease development. Additionally, we have provided the first data suggesting that distinct early interactions of phagocytes with *S*. Typhi might be critical for disease control. Activation of appropriate signaling pathways might lead to effective activation of the cells which will ultimately limit bacterial infection, thereby avoiding disease. It is unlikely that monocytes/macrophages and DCs are the sole contributors to this phenomenon. A constellation of other innate and adaptive immune responses might also contribute to limit the disease; however, the mechanisms by which development of disease is controlled remain to be explored.

## Supporting Information

S1 Fig Percentage of monocytes in whole blood and expression of integrin α4β7 by DCs following challenge with wt *S*. Typhi. Shown in panels A and B are the percentages of monocytes in whole blood as determined by WCC (whole blood cell counts). These measurements were obtained as part of routine blood hematology and biochemistry evaluations performed on alternate days after challenge and at typhoid diagnosis. A routine haematology cytometer was used to measure the total and differential white cell count in the samples collected from the participants. Tests were run in the hospital clinical laboratory according to local and national Standard Operating Procedures (SOPs) and with regular quality control (QC) standardization. The measurements obtained in the clinical laboratory have already been published (Waddington CS, et al., Clinical Infectious Diseases, 2014). Panels C and D show kinetics of the expression of integrin α4β7 by DCs in TD and NoTD volunteers, respectively.
(DOCX)

S2 Fig Evaluation of multi-marker expression by monocytes and DCs at TD 48h. To determine the dependence of upregulation of activation markers on the binding capacity to *S*. Typhi, boolean gates for all the evaluated markers were used. For this analysis, the percentage of cells binding to *S*. Typhi was determined using gates based on FMO stainings. Data are presented as % net change as related to baseline levels (pre-challenge) and represented in box and whisker (90–10 percentile) plots. Only the five most dominant populations (of 16 possible combinations) are displayed for both monocytes and DCs. White bars indicate populations that bound to *S*. Typhi (*S*. Typhi^+^); while gray bars indicate the ones that did not (*S*. Typhi^-^). * p < 0.05 (Bonferroni’s multiple comparison test). # p<0.05 comparison between *S*. Typhi binding and non-binding groups (Mann Whitney test).
(DOCX)

S3 Fig Monocytes and DCs show different CD40 up-regulation kinetics. In TD volunteers both monocytes and DCs up-regulated CD40 during typhoid disease days. However, the up-regulation kinetics for this marker appeared slightly different since in DCs (open squares) CD40 up-regulation peaked at TD-0h, while in monocytes (closed circles) maximum upregulation was observed 2 days later (TD-48h). No statistically significant differences were observed in the percentages of monocytes and DCs at peak times (Monocytes vs DCs).
(DOCX)

S4 Fig Markers used to confirm identity of DCs. The identity of DCs was confirmed by the lack of expression of lineage markers (CD3, CD56, CD66b) and expression of CD123 and CD11c (shown in Fig 1). To further confirm the identity of DCs, expression of BDCA-1, BDCA-2 and BDCA-3 were evaluated in CD14^-^ HLA-DR^+^ cells. As controls for expression of the previously mentioned markers, fluorescent minus one (FMO) for each marker was included in all experiments (gray histograms or indicated as FMOs). Displayed are plots from a representative volunteer.
(DOCX)
