# Supplementary material for: Oral Wild-Type Salmonella Typhi Challenge Induces Activation of Circulating Monocytes and Dendritic Cells in Individuals Who Develop Typhoid Disease
Source: PLoS Negl Trop Dis. 2015 Jun 11;9(6):e0003837. doi: 10.1371/journal.pntd.0003837 (PMC4465829; doi:10.1371/journal.pntd.0003837)
Supplement: S1 Fig — Shown in panels A and B are the percentages of monocytes in whole blood as determined by WCC (whole blood cell counts). These measurements were obtained as part of routine blood hematology and biochemistry evaluations performed on alternate days after challenge and at typhoid diagnosis. A routine haematology cytometer was used to measure the total and differential white cell count in the samples collected from the participants. Tests were run in the hospital clinical laboratory according to local and national Standard Operating Procedures (SOPs) and with regular quality control (QC) standardization. The measurements obtained in the clinical laboratory have already been published (Waddington CS, et al., Clinical Infectious Diseases, 2014). Panels C and D show kinetics of the expression of integrin α4β7 by DCs in TD and NoTD volunteers, respectively. (DOCX) [file pntd.0003837.s001.docx]

**S1 Fig. Percentage of monocytes in whole blood and expression of integrin α4β7 by DCs following challenge with wt *S*. Typhi.** Shown in panels A and B are the percentages of monocytes in whole blood as determined by WCC (whole blood cell counts). These measurements were obtained as part of routine blood hematology and biochemistry evaluations performed on alternate days after challenge and at typhoid diagnosis. A routine haematology cytometer was used to measure the total and differential white cell count in the samples collected from the participants. Tests were run in the hospital clinical laboratory according to local and national Standard Operating Procedures (SOPs) and with regular quality control (QC) standardization. The measurements obtained in the clinical laboratory have already been published [1]. Panels C and D show kinetics of the expression of integrin α4β7 by DCs in TD and NoTD volunteers, respectively.

**References**

1. Waddington CS, Darton TC, Jones C, Haworth K, Peters A, John T, et al. An Outpatient, Ambulant-Design, Controlled Human Infection Model Using Escalating Doses of Salmonella Typhi Challenge Delivered in Sodium Bicarbonate Solution. Clinical Infectious Diseases. 2014;58(9):1230-40.
