# Supplementary material for: Oral Wild-Type Salmonella Typhi Challenge Induces Activation of Circulating Monocytes and Dendritic Cells in Individuals Who Develop Typhoid Disease
Source: PLoS Negl Trop Dis. 2015 Jun 11;9(6):e0003837. doi: 10.1371/journal.pntd.0003837 (PMC4465829; doi:10.1371/journal.pntd.0003837)
Supplement: S2 Fig — To determine the dependence of upregulation of activation markers on the binding capacity to S. Typhi, boolean gates for all the evaluated markers were used. For this analysis, the percentage of cells binding to S. Typhi was determined using gates based on FMO stainings. Data are presented as % net change as related to baseline levels (pre-challenge) and represented in box and whisker (90–10 percentile) plots. Only the five most dominant populations (of 16 possible combinations) are displayed for both monocytes and DCs. White bars indicate populations that bound to S. Typhi (S. Typhi+); while gray bars indicate the ones that did not (S. Typhi-). * p < 0.05 (Bonferroni’s multiple comparison test). # p<0.05 comparison between S. Typhi binding and non-binding groups (Mann Whitney test). (DOCX) [file pntd.0003837.s002.docx]

**S2 Fig. Evaluation of multi-marker expression by monocytes and DCs at TD 48h.** To determine the dependence of upregulation of activation markers on the binding capacity to *S*. Typhi, boolean gates for all the evaluated markers were used. For this analysis, the percentage of cells binding to *S.* Typhi was determined using gates based on FMO stainings. Data are presented as % net change as related to baseline levels (pre-challenge) and represented in box and whisker (90-10 percentile) plots. Only the five most dominant populations (of 16 possible combinations) are displayed for both monocytes and DCs. White bars indicate populations that bound to *S*. Typhi (*S*. Typhi^+^); while gray bars indicate the ones that did not (*S*. Typhi^-^). * p < 0.05 (Bonferroni’s multiple comparison test). # p<0.05 comparison between *S*. Typhi binding and non-binding groups (Mann Whitney test).
