# Supplementary material for: Oral Wild-Type Salmonella Typhi Challenge Induces Activation of Circulating Monocytes and Dendritic Cells in Individuals Who Develop Typhoid Disease
Source: PLoS Negl Trop Dis. 2015 Jun 11;9(6):e0003837. doi: 10.1371/journal.pntd.0003837 (PMC4465829; doi:10.1371/journal.pntd.0003837)
Supplement: S3 Fig — In TD volunteers both monocytes and DCs up-regulated CD40 during typhoid disease days. However, the up-regulation kinetics for this marker appeared slightly different since in DCs (open squares) CD40 up-regulation peaked at TD-0h, while in monocytes (closed circles) maximum upregulation was observed 2 days later (TD-48h). No statistically significant differences were observed in the percentages of monocytes and DCs at peak times (Monocytes vs DCs). (DOCX) [file pntd.0003837.s003.docx]

**S3 Fig. Monocytes and DCs show different CD40 up-regulation kinetics.** In TD volunteers both monocytes and DCs up-regulated CD40 during typhoid disease days. However, the up-regulation kinetics for this marker appeared slightly different since in DCs (open squares) CD40 up-regulation peaked at TD-0h, while in monocytes (closed circles) maximum up-regulation was observed 2 days later (TD-48h). No statistically significant differences were observed in the percentages of monocytes and DCs at peak times (Monocytes vs. DCs).
