# Supplementary material for: Oral Wild-Type Salmonella Typhi Challenge Induces Activation of Circulating Monocytes and Dendritic Cells in Individuals Who Develop Typhoid Disease
Source: PLoS Negl Trop Dis. 2015 Jun 11;9(6):e0003837. doi: 10.1371/journal.pntd.0003837 (PMC4465829; doi:10.1371/journal.pntd.0003837)
Supplement: S4 Fig — The identity of DCs was confirmed by the lack of expression of lineage markers (CD3, CD56, CD66b) and expression of CD123 and CD11c (shown in Fig 1). To further confirm the identity of DCs, expression of BDCA-1, BDCA-2 and BDCA-3 were evaluated in CD14- HLA-DR+ cells. As controls for expression of the previously mentioned markers, fluorescent minus one (FMO) for each marker was included in all experiments (gray histograms or indicated as FMOs). Displayed are plots from a representative volunteer. (DOCX) [file pntd.0003837.s004.docx]

**S4 Fig.** **Markers used to confirm identity of DCs.** The identity of DCs was confirmed by the lack of expression of lineage markers (CD3, CD56, CD66b) and expression of CD123 and CD11c (shown in Fig 1). To further confirm the identity of DCs, expression of BDCA-1, BDCA-2 and BDCA-3 were evaluated in CD14- HLA-DR+ cells. As controls for expression of the previously mentioned markers, fluorescent minus one (FMO) for each marker was included in all experiments (gray histograms or indicated as FMOs). Displayed are plots from a representative volunteer.

**
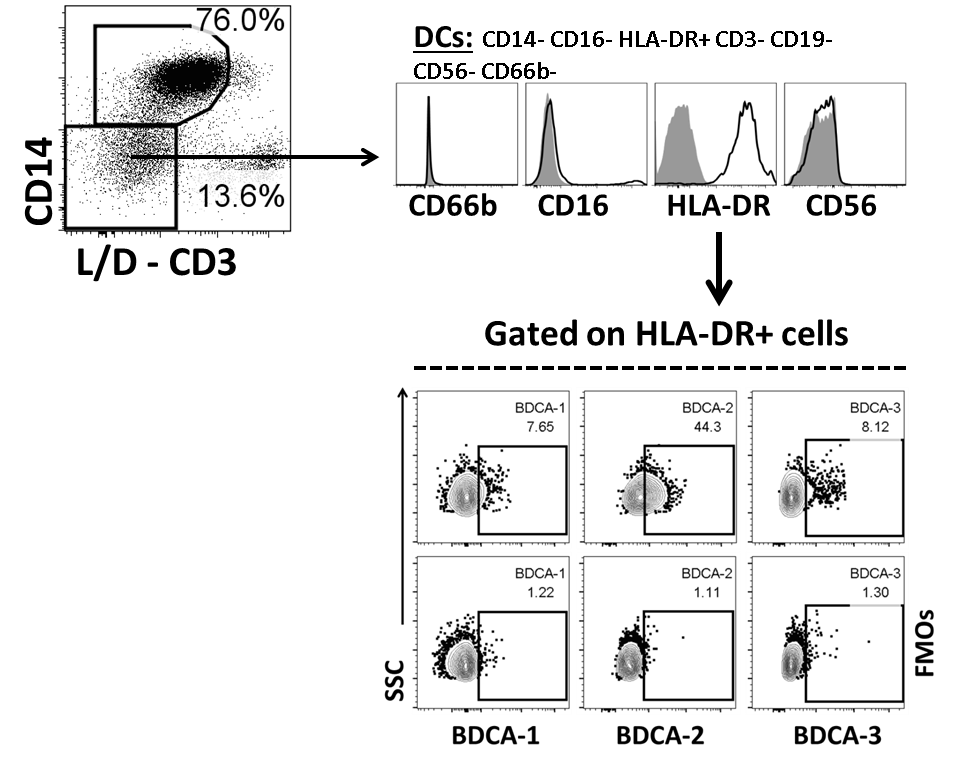
**
